# KAI2 Can Do: Karrikin Receptor Function in Plant Development and Response to Abiotic and Biotic Factors

**DOI:** 10.1093/pcp/pcad077

**Published:** 2023-07-19

**Authors:** Kartikye Varshney, Caroline Gutjahr

**Affiliations:** Department of Root Biology and Symbiosis, Max Planck Institute of Molecular Plant Physiology, Potsdam Science Park, Am Mühlenberg 1, Potsdam-Golm 14476, Germany; Department of Root Biology and Symbiosis, Max Planck Institute of Molecular Plant Physiology, Potsdam Science Park, Am Mühlenberg 1, Potsdam-Golm 14476, Germany

**Keywords:** Karrikin signaling, Plant development, Plant hormones, Plant–microbe interaction, Stress response

## Abstract

The α/β hydrolase KARRIKIN INSENSITIVE 2 (KAI2) functions as a receptor for a yet undiscovered phytohormone, provisionally termed KAI2 ligand (KL). In addition, it perceives karrikin, a butenolide compound found in the smoke of burnt plant material. KAI2-mediated signaling is involved in regulating seed germination and in shaping seedling and adult plant morphology, both above and below ground. It also governs responses to various abiotic stimuli and stresses and shapes biotic interactions. KAI2-mediated signaling is being linked to an elaborate cross-talk with other phytohormone pathways such as auxin, gibberellin, abscisic acid, ethylene and salicylic acid signaling, in addition to light and nutrient starvation signaling. Further connections will likely be revealed in the future. This article summarizes recent advances in unraveling the function of KAI2-mediated signaling and its interaction with other signaling pathways.

## Introduction

Karrikins (KARs) are butenolide compounds that were first identified as active components of smoke from burnt plant material that can stimulate the germination of dormant seeds from fire-following plants (Flematti et al. 2004). Critical for understanding KAR perception was the observation that KARs can trigger seed germination and seedling growth responses also in model plants such as *Arabidopsis thaliana*, which do usually not experience fire (Nelson et al. 2009). Reverse genetics in *Arabidopsis* identified *KARRIKIN INSENSITIVE 2* (*KAI2*), encoding an α/β hydrolase protein, as being required for KAR-mediated seed germination (Waters et al. 2012). Subsequently, evidence from in vitro binding assays and co-crystallization of a protein-ligand complex suggested that KAI2 may have the ability to perceive KARs (Guo et al. 2013, Kagiyama et al. 2013). However, circumstantial evidence suggests that KAR may have to be metabolized before it can be perceived (Waters et al. 2015a, Sepulveda et al. 2022). Importantly, developmental phenotypes in the absence of fire and marker gene responses to *Arabidopsis* extracts suggest that KAI2 perceives an endogenous molecule provisionally termed KAI2 ligand (KL) (Conn and Nelson 2015, Sun et al. 2016) which likely represents a phytohormone. Fourteen years since the first description of smoke-derived KARs and 11 years after the identification of the receptor KAI2, the identity of KL remains elusive.

Signal transduction via KAI2 requires an F-box protein from the Skp, Cullin, F-box (SCF)-type E3 ubiquitin ligase complex called MORE AXILLIARY GROWTH 2 (MAX2) (Waters et al. 2012). Activation of KAI2 in the presence of SCF^MAX2^ leads to ubiquitination and subsequent degradation of KAR response–specific members of the SUPPRESSOR OF MAX2-LIKE (SMXL) protein family (SMAX1 and SMXL2) (Carbonnel et al. 2020a, Khosla et al. 2020, Wang et al. 2020, Zheng et al. 2020). *SMAX1* was discovered in a suppressor screen for KAR signaling–related *max2* phenotypes revealing its role as a negative regulator of KAR signaling (Stanga et al. 2013).

*MAX2* is also involved in signal transduction upon perception of carotenoid-derived plant hormones called strigolactones (SLs) (Nelson et al. 2011, Wang et al. 2022), and *max2* mutants show both KAR- and SL-related phenotypes. SLs are perceived by the KAI2-related receptor DWARF14 (D14) which together with SCF^MAX2^ leads to the degradation of SL response–specific members of the SMXL protein family (SMXL6,7,8 in *Arabidopsis* and D53 in rice) (reviewed in Kyozuka et al. 2022). The use of *max2* to study SL responses has led to the mischaracterization of some of its KAR outputs as SL effects, highlighting the need to use specific *kai2* and *d14* mutants (Scaffidi et al. 2014, Villaécija-Aguilar et al. 2019). Furthermore, the synthetic SL analog *rac*-GR24 routinely used to induce SL-dependent responses contains two stereoisomers, of which GR24^5DS^ specifically activates D14, while the other GR24*^ent^*^-5DS^ activates KAI2 (Scaffidi et al. 2014).

Here, we focus on KAR/KL signaling and review the current knowledge on the biological roles of KAI2 in plant development, stress tolerance and interaction with microorganisms. Additionally, we highlight that KAI2 is an indispensable player in the intricate interlacing of phytohormone signaling pathways.

## KAI2 Regulates Multiple Aspects of Plant Growth and Development

### Germination

**Fig. 1:**
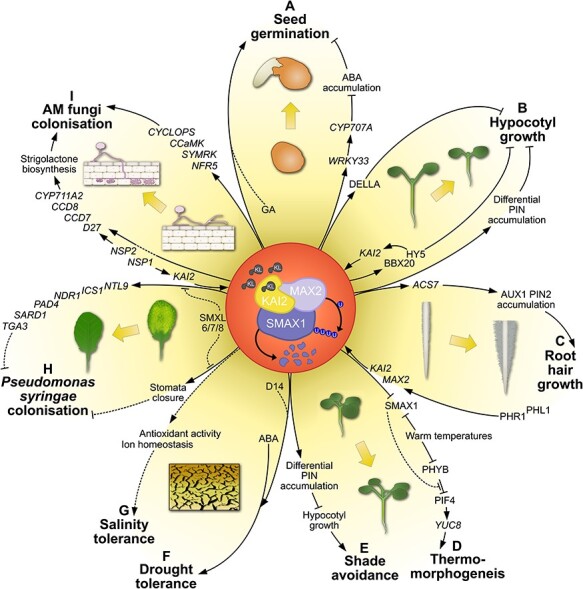
Mechanisms of KAI2-mediated plant development and response to abiotic and biotic factors. KAI2, an α/β hydrolase receptor in the KAR-signaling pathway, governs multiple aspects of plant development and abiotic and biotic interactions. On perception of a predicted phytohormone, tentatively called KL, KAI2 forms a complex with the F-box protein MAX2, leading to degradation of SMAX1 and thus activating downstream responses (Stanga et al. 2013, Carbonnel et al. 2020a, Khosla et al. 2020, Zheng et al. 2020). (A) During germination of *Arabidopsis* seeds, KAI2 signaling leads to the induction of *WRKY33* which in turn induces *CYP707A*, encoding an ABA catabolic enzyme, thereby reducing ABA-induced seed dormancy (Brun et al. 2019). A partial dependence on canonical GA signaling has also been observed, indicating that KAI2 signaling likely acts on seed germination by modulating the balance of ABA vs. GA signaling (Nelson et al. 2009). (B) In hypocotyl development, SMAX1 degradation results in the accumulation of DELLA which negatively regulates hypocotyl growth (Kim et al. 2022). KAI2 also modulates the abundance of PIN proteins in the hypocotyl to facilitate auxin movement away from the hypocotyl resulting in growth reduction (Hamon-Josse et al. 2022). Additionally, KAI2 influences the cessation of hypocotyl growth in the dark-to-light transition via *HY5* and affects transcriptional induction and post-translation stabilization of the HY5 interactor BBX20. Furthermore, HY5 binds to the *KAI2* promoter and regulates *KAI2* induction upon transition from the dark to light in a possible feed-forward loop (Sun and Ni 2011, Waters and Smith 2013, Bursch et al. 2021). (C) PHR1 and PHL1 (transcription factors regulating phosphate starvation responses; Paries and Gutjahr 2023) are required for induction of *KAI2* and *MAX2* in response to phosphate deficiency (Villaécija-Aguilar et al. 2022). Activation of KAI2 signaling induces *ACS7* expression leading to increased ethylene biosynthesis (Carbonnel et al. 2020a). This leads to AUX1 and PIN2 accumulation in specific regions of the root tip, optimizing auxin levels in root tip epidermal cells to drive root hair growth (Villaécija-Aguilar et al. 2022). (D) High ambient temperatures inactivate PHYB thereby releasing PIF4 repression which coordinates thermomorphogenic hypocotyl growth via inducing the auxin biosynthesis gene *YUC8* (Sun et al. 2012, Jung et al. 2016). SMAX1 somehow blocks the repression of PIF4 by PHYB, positively regulating hypocotyl growth. Excess growth is kept in check by SMAX1 destabilization on long exposure to warm temperatures (Park et al. 2022). (E) Hypocotyl growth contributes to shade avoidance response (Ciolfi et al. 2013). KAI2 signaling negatively regulates shade-induced hypocotyl growth by influencing the accumulation of PIN proteins to move auxin away from the hypocotyl (Xu et al. 2022). (F) KAI2 signaling positively influences drought tolerance response by modulating plant morphology for reduced water loss under stress, regulating ROS accumulation and enhancing ABA responsiveness (Li et al. 2017, Feng et al. 2023). These responses are co-regulated by D14 albeit with varying levels of D14 contribution (Li et al. 2017, 2020). (G) KAI2 signaling influences the expression of genes involved in maintaining ion homeostasis and ROS levels in plant cells which could be the cause of enhanced salinity tolerance linked to KAI2 (Mostofa et al. 2023). (H) KAI2 plays a role in defense against the hemibiotrophic bacterium *Pseudomonas syringae* pv. *tomato*. Normal induction of genes involved in salicylic acid–dependent resistance under pathogen stress such as *NTL9*, *ICS1*, *NDR1*, *PAD4*, *SARD1* and *TGA3* is dependent on functional KAI2 signaling. Additionally, stomatal closure to prevent pathogen entry also requires KAI2. The formation of a complex of SMAX1 with SMAX1-LIKE 6/7/8 is predicted to regulate the defense response (Zheng et al. 2023). (I) Expression of key genes of the common symbiosis signaling network, promoting colonization by AM fungi in rice, such as *NFR5*, *SYMRK*, *CCaMK* and *CYCLOPS*, is promoted upon SMAX1 removal (Choi et al. 2020). *NODULATION SIGNALING PATHWAY 1* (*NSP1*) and *NSP2* are transcription factors inducing SL biosynthesis genes such as *D27*, *CCD7*, *CCD8* and *CYP711A2* (Liu et al. 2011, Zhang et al. 2014). The resulting enhanced root exudation of SL promotes root colonization by AM fungi. This promotion by NSP1 and NSP2 requires functional *KAI2* (Li et al. 2022b). KAI2 signaling also regulates the expression of SL biosynthesis genes (Choi et al. 2020); however, whether this happens via the NSPs is not yet clear.

Consistent with the role of KARs in inducing seed germination, mutations in *KAI2* result in enhanced primary dormancy of *Arabidopsis* seeds (Waters et al. 2012). Gibberellins (GAs) and abscisic acid (ABA) are major and antagonistic regulators promoting seed germination and dormancy, respectively (Finch-Savage and Leubner-Metzger 2006). The *htl-3* mutant (mutated in *KAI2*) is impaired in *rac*-GR24-mediated induction of a key ABA catabolism gene *CYP707A* and the gene encoding the transcription factor WRKY33, which regulates *CYP707A* expression, possibly resulting in ABA accumulation and promotion of dormancy (**Fig. 1A**). However, *wrky33* and *cyp707a* mutants partially retain germination responses to *rac*-GR24, indicating that these genes are not the sole target of KAI2 signaling (Brun et al. 2019).

The promotion of seed germination upon KAR treatment requires GA biosynthesis. But KAR_1_ partially induces germination in the GA signaling mutant *sleepy* (*sly*) suggesting only partial dependence on canonical GA signaling (Nelson et al. 2009) (**Fig. 1A**). Interestingly, *Arabidopsis smax1* seeds germinate even in the presence of GA biosynthesis inhibitors, suggesting that SMAX1 may be required for suppression of germination by DELLA proteins, the proteolytic targets (and repressors) of GA signaling (Bunsick et al. 2020).

### Hypocotyl growth

KAI2 signaling regulates the inhibition of hypocotyl growth, e.g. during photomorphogenesis. *Arabidopsis kai2* seedlings show an elongated hypocotyl compared to the wild type in dark-to-light transitions but not in continuous darkness, as well as a reduced cotyledon size (Sun and Ni 2011, Waters et al. 2012). In turn, *smax1 smxl2* double mutants show shorter hypocotyls and larger cotyledons than wild-type plants, similar to the wild type after KAR treatment (Stanga et al. 2016). How KAI2 signaling suppresses hypocotyl elongation exactly, is not yet known. There are first indications that the interaction with other phytohormone signaling pathways plays a role in this process.

KAI2 signaling regulating hypocotyl elongation appears to be partially controlled by light. Seedlings grown in the dark after germination show an increase in *KAI2* expression when transferred to red light. This short-term increase depends on *HY5*, encoding a transcription factor that positively regulates responses to light (Sun and Ni 2011, Waters and Smith 2013). However, wild-type and *hy5* seedlings grown in continuous darkness or continuous light display the same *KAI2* transcript accumulation, suggesting that HY5 is only required for *KAI2* induction during the dark-to-light transition (Waters and Smith 2013). The hypocotyl response to KAR treatment is partially impaired in *hy5* mutants (Nelson et al. 2010, Waters and Smith 2013), while *kai2* and *max2* mutations have additive effects to *hy5* (Waters and Smith 2013). This suggests that HY5 regulates pathways that suppress hypocotyl elongation in parallel with KAI2 signaling. Additionally, members of the BBX zinc finger family in *Arabidopsis*, which interact with HY5 and positively regulate photomorphogenic responses, are partially required for KAI2-mediated hypocotyl reduction (Bursch et al. 2021) (**Fig. 1B**).

Kim et al. (2022) demonstrated that the dark-to-light transition is accompanied by accumulation of SMAX1 in *Arabidopsis* seedlings, while in the dark, SMAX1 accumulation is low and decreases with time. This suggests that KAI2 signaling plays a more important role in hypocotyl growth regulation in the light than in the dark. In the light, SMAX1 supports hypocotyl elongation by preventing accumulation of DELLA proteins in hypocotyl cell nuclei (Kim et al. 2022). Light-mediated inhibition of hypocotyl growth is hampered in DELLA-deficient mutants, while light exposure leads to a decrease in endogenous GA levels and subsequent DELLA accumulation (Achard et al. 2007). KAR treatment results in light and KAI2 signaling–dependent accumulation of DELLA proteins in hypocotyl cells resulting in cessation of growth (Kim et al. 2022) (**Fig. 1B**). Thus, additional environmental stimuli or the composition of the light spectrum may regulate the production of KL and thereby the level of KAI2 activity, SMAX1 degradation, DELLA accumulation and hypocotyl growth. In addition, independent pathways may influence SMAX1 stability and thereby hypocotyl elongation (Khosla et al. 2020).

During the dark-to-light transition, KAI2 also modulates auxin signaling. It likely facilitates the rapid movement of auxin produced at the shoot apex toward the root meristem by fine-tuning the abundance of auxin transport proteins of the PIN family (Hamon-Josse et al. 2022) (**Fig. 1B**). Reduction in PIN3, PIN4 and PIN7 abundance in the hypocotyl and an increase in PIN1, PIN3 and PIN7 abundance at the root meristem are thought to stall hypocotyl elongation while promoting root growth. This remodeling of PIN abundance is absent from *kai2* mutants resulting in auxin build-up in the hypocotyl causing an increase in hypocotyl length.

Interestingly, for *kai2a kai2b* double mutants of the model legume *Lotus japonicus* no hypocotyl phenotype has been observed, indicating differences in the role of KAI2 in this process among species (Carbonnel et al. 2020b) In rice, mesocotyl elongation in the dark is controlled by D14L (encoded by the rice ortholog of *AtKAI2*) and D14 in an additive manner (Gutjahr et al. 2015, Kameoka and Kyozuka 2015, Zheng et al. 2020). Interestingly, mutation of *smax1* in rice suppresses the long mesocotyl phenotype of *d14* and *d14l* alike (Choi et al. 2020, Zheng et al. 2020), suggesting that both D14 and D14L target SMAX1 in the context of hypocotyl elongation. Indeed, also in *Arabidopsis*, D14 can target SMAX1 upon *rac*-GR24 treatment (Li et al. 2022a). However, not only SMAX1 but also D53 seems capable of promoting rice mesocotyl elongation as the rice *d53* mutant, which expresses a degradation-resistant D53 has similarly long mesocotyls as *d14* (Zheng et al. 2020). A *d53* knockout mutant was to our knowledge not tested. Together, this suggests that SMAX1 can be targeted by both D14L and D14. Furthermore, SMAX1 and D53 may be able to interact with the same target proteins in the context of mesocotyl growth, but it is possible that wild-type D53 does not normally accumulate in the tissues relevant for mesocotyl elongation (*d53* knockout mutant analysis is necessary to investigate that).

### Leaf and shoot development

Above-ground morphological phenotypes of *Arabidopsis kai2* mutants at a later stage of development include elongated petioles and increased blade length and width contrary to the effects of *d14* (Sun and Ni 2011, Waters et al. 2012, Soundappan et al. 2015, Bennett et al. 2016). Thus, the ratio of *KAI2* and SL signaling potentially regulates mature leaf morphology in *Arabidopsis*.

*Brachypodium distachyon kai2* mutants exhibit longer internodes resulting in an elongated appearance (Meng et al. 2022). Green revolution involved the integration of dwarfing traits in crops which facilitated bearing heavy grain without experiencing yield loss due to lodging. The genes for these traits regulate GA production or sensitivity (Hedden 2003). Given that there seems to be an interaction between KAI2-mediated signaling and GA signaling, it is possible that GA plays a role in promoting internode growth in *Brachypodium kai2*. Currently, there is no report of this phenotype from other plant species, and no molecular data explaining the phenotype are available.

### Root and root hair development

Roots and root hairs help in anchorage and acquisition of water and mineral nutrients from the soil. Negative gravitropism ensures that roots grow straight down but additional directional regulation is necessary to maximize nutrient retrieval while avoiding potential stressors such as obstacles, excess salt or dry soil patches (Su et al. 2017). KAI2 regulates multiple aspects of root system architecture including primary root length, root skewing and waving on agar surfaces, emergence of adventitious and lateral roots, lateral root number and root hair length and density with some variations among species (Swarbreck et al. 2019, 2020, Villaécija-Aguilar et al. 2019, 2022, Carbonnel et al. 2020a, b, Hamon-Josse et al. 2022, Meng et al. 2022).

*Arabidopsis kai2* mutants show a decreased root hair length and density (Villaécija-Aguilar et al. 2019). This phenotype is conserved in *B. distachyon kai2* and *BdKAI2* can restore root hair growth in *Arabidopsis kai2* (Meng et al. 2022). Interestingly, root hairs of *Lotus japonicus* and pea *kai2a kai2b* double mutants (in genomes of these legumes *KAI2* is duplicated) have the same length as wild-type root hairs (Carbonnel et al. 2020a, Guercio et al. 2022). However, *smax1* mutants of *L. japonicus* show an increase in root hair length like the *smax1 smxl2* double mutant of *Arabidopsis* (Villaécija-Aguilar et al. 2019, Carbonnel et al. 2020a). Similarly, *Brachypodium kai2* shows an increase in the distance of the emergence of first root hairs from the quiescent center, but this is not observed for the *Lotus* mutants. However, *Lotus smax1* shows a decrease in this distance (Carbonnel et al. 2020a, Meng et al. 2022). The conservation of *smax1* root hair phenotypes but the absence of *kai2* root hair phenotypes in *L. japonicus* and pea suggests that in these legumes redundant factors may influence the stability or activity of SMAX1.

Carbonnel et al. (2020a) and Villaécija-Aguilar et al. (2022) described the molecular mechanism of *KAI2*-mediated-signaling in root system and root hair development. Carbonnel et al. (2020a) demonstrated that in *L. japonicus*, SMAX1 negatively regulates the expression of *1-AMINOCYCLOPROPANE CARBOXYLIC ACID* (ACC) *SYNTHASE 7* (*ACS7*), involved in ethylene biosynthesis, to regulate root system architecture. *smax1* mutants produce higher amounts of ethylene than the wild type and exhibit increased expression of *ACS7* causing longer root hairs and shorter primary roots. The phenotypes could be fully rescued by treating with the ethylene biosynthesis inhibitor 2-aminoethoxyvinyl glycine and the ethylene signaling inhibitor, AgNO_3_ (Schaller and Binder 2017, Carbonnel et al. 2020a). Congruously, the short root hair phenotype of *Arabidopsis kai2* and *acs7* mutants was rescued by treatment with the ethylene precursor ACC (Villaécija-Aguilar et al. 2022). *KAI2* is also required for accumulation of the auxin importer AUXIN TRANSPORTER PROTEIN 1 (AUX1) in the epidermis above the lateral root cap (LRC), the LRC, the root tip and the stele; and of the auxin exporter PIN-FORMED2 (PIN2) in the meristematic zone of the root tip. This seems to optimize the auxin level in epidermal cells above the LRC, promoting root hair elongation. Furthermore, *AUX1* and *PIN2* are required for KAR- and ACC-mediated root hair elongation, placing them downstream of KAR as well as ethylene signaling. Thus, a *KAI2*-ethylene-auxin-signaling cascade commands root hair growth in *Arabidopsis* (Villaécija-Aguilar et al. 2022) (**Fig. 1C**).

Lateral root formation differs from other root traits in *Arabidopsis*, as it is regulated by both *KAI2* and *D14* in an additive manner with both mutants showing increased lateral root density (Villaécija-Aguilar et al. 2019). Formation of junction roots, a type of adventitious roots in *Arabidopsis*, is also under the control of both *KAI2* and *D14* signaling but with a stronger impact of *KAI2* (Swarbreck et al. 2020). In the grass *B. distachyon*, mutation of *KAI2* alone leads to a dramatic increase in lateral roots (Meng et al. 2022), but the role of *D14* has not been investigated. Rice *d14l* seems to have increased large lateral root density, but the significance of this difference to the wild type was not determined (Chiu et al. 2018). Interestingly, *L. japonicus kai2a kai2b* mutants do not show an increase in lateral roots (a difference to the wild type only becomes apparent upon karrikin treatment, Carbonnel et al. 2020b) but the *smax1* mutant does (Carbonnel et al. 2020a). This, along with differences in root hair phenotypes compared to other plants, suggests diversification of KAI2 function in *L. japonicus* in defining root system architecture or variation in nutritional optima among species, which would shift the role of KAI2 to either positive or negative for lateral root development.

### Role of KAI2 in bryophyte development

*KAI2* orthologs can be traced back to charophyte algae (Delaux et al. 2012) and possibly arose via horizontal gene transfer of bacterial *RsbQ* (Wang et al. 2022). It is not yet clear which function KAI2 performs in algae. By contrast, the role of KAI2 in bryophytes is beginning to be revealed. During the early evolution of land plants, the *KAI2* lineage split, giving rise to two superclades, the *eu-KAI2* clade containing the characterized *KAI2* sequences from angiosperms and homologs from other land plants and the *DDK* (*D14*/*DLK2*/*KAI2*) clade containing SL receptor *D14* homologs, divergent bryophyte *KAI2*s and *D14-LIKE 2* (*DLK2*) homologs (Bythell-Douglas et al. 2017). In the moss *Physcomitrium patens, eu-KAI2* mutants are smaller than the wild type but have bigger gametophores. Additionally, they have longer gametophores when grown under continuous red light pointing to a role of KAI2 in photomorphogenesis (Lopez-Obando et al. 2021). These phenotypes are similar to that of *Ppmax2* suggesting MAX2-dependent signal transduction via PpKAI2L (eu-KAI2) (Lopez-Obando et al. 2018). Interestingly, other homologs of *PpKAI2L* function in a *MAX2*-independent but *PpCCD8*-dependent manner, thereby regulating SL-specific responses (Lopez-Obando et al. 2021). This suggests that neo-functionalization of KAI2 to perceive SLs occured independently in mosses (Lopez-Obando et al. 2016).

The liverwort *Marchantia polymorpha* has two *eu-KAI2* orthologs, *MpKAI2a* and *MpKAI2b*, and one each of *MpMAX2* and *MpSMXL* (Waters et al. 2012, Mizuno et al. 2021). Phenotypic analysis of *Mpkai2* mutants revealed morphological defects including reduced thallus area, increased angle of thallus curving, reduced gemma area and gemma formation and reduced number of cells in gemma. Mutants were also impaired in photomorphogenic responses and showed uninhibited growth in the dark. MpKAI2a and MpKAI2b show possible physical interactions with only GR24*^ent^*^-5DS^ but they do not respond to KARs (with only *MpKAI2A* seeming to regulate downstream responses) (Mizuno et al. 2021, Komatsu et al. 2023), indicating that KL is different from KARs found in smoke, at least in *Marchantia*.

*Mpsmxl* mutation can fully suppress the thallus angle, the gemma cell number and partially the thallus area phenotype. However, it shows the same reduction in the gemma area as *Mpkai2a* (Mizuno et al. 2021), suggesting that this trait is regulated by KAI2 in an SMXL-independent fashion. In fact, *SMXL* genes first appeared in mosses and liverworts and later duplicated and diversified in synchrony with the diversification of the receptor proteins (Moturu et al. 2018, Walker et al. 2019). Thus, in charophytes, KAI2 must have functioned independently of SMXLs and it seems that remnant SMXL-independent functions still exist in land plants.

## Roles of KAI2 in Abiotic Stress Responses

In recent years, evidence is accumulating that KAI2 signaling not only regulates plant development per se but especially as a reaction to environmental conditions.

### Heat tolerance

Global surface temperatures tend to fluctuate due to various factors but there has been an unusual trend toward warming in the last 20 years (Neukom et al. 2019). Plants deploy various mechanisms to evade serious consequences from an increase in ambient temperatures beyond the optimum (Wang et al. 2017, Wen et al. 2019) and display developmental responses to elevated temperatures, a phenomenon called thermomorphogenesis (Delker et al. 2022).

When *Arabidopsis* seeds are exposed to high ambient temperatures (30°C) for 4 d and transferred back to optimal conditions (20°C), *kai2* seeds experience a reduced germination success compared to wild-type seeds and compared to *kai2* seeds continuously incubated at 20°C (Wang et al. 2018). Wild-type seeds recover very well after high-temperature stress suggesting a positive role of *KAI2* in heat tolerance. Interestingly, treatment with KAR_2_ strongly inhibits seed germination in the wild type at higher temperatures. With the role of KAI2 in regulating seed dormancy already established, Wang et al. (2018) argued that in wild-type seeds, KAI2 could promote seed germination in favorable conditions but prevent seed germination in unfavorable conditions. This is conceivable under the assumption that SMAX1-interacting proteins change depending on the environmental condition (e.g. by their availability), and SMAX1 may block promoters of germination under favorable conditions and inhibitors of germination under unfavorable conditions.

*KAI2* is also required for high-temperature tolerance post-germination. Ten-day-old *Arabidopsis kai2* plants when exposed to 40°C for 5 d show higher mortality rates than wild type. This is accompanied by compromised cell membrane integrity and inability to regulate leaf surface temperature in *kai2* (Abdelrahman et al. 2023). Plants tend to close stomata in events of high-temperature stress to not excessively lose water (Marchin et al. 2022). It has previously been shown in *Arabidopsis kai2* that stomatal apertures are larger which can possibly exacerbate the effects of heat (Li et al. 2017, Marchin et al. 2022). Transcriptional data analysis revealed lower expression of a battery of genes in *kai2* which are involved in plant thermo-tolerance. This included heat-shock protein-related genes like *HSP70, HSP90* and *HSP101*, membrane fluidity thermosensors of *CYCLIC NUCLEOTIDE GATED CHANEL* gene family and *WRKY* transcription factors among others (Abdelrahman et al. 2023).

*Arabidopsis* KAI2-signaling mutants, when exposed to high-temperature stress of 28°C, have impaired thermomorphogenic hypocotyl growth, with *kai2* having a longer and *smax1* having a shorter hypocotyl compared to the wild type under stress (Park et al. 2022). PHYTOCHROME B (PHYB) has been established as a thermosensor that negatively regulates PHYTOCHROME-INTERACTING FACTOR 4 (PIF4) activity in promoting hypocotyl growth in response to high-temperature stress (Jung et al. 2016). Interestingly, the expression of a constitutively active *phyB* in *kai2* or *max2* background reduces thermomorphogenic hypocotyl growth, while inactive *phyB* is epistatic to *smax1* (Park et al. 2022). Furthermore, *Arabidopsis pif4* can suppress hypocotyl growth in *kai2*, whereas supplementary expression of the wild type version can rescue the attenuated response of *smax1*. This suggests that the PHYB-PIF4 module acts downstream to KAI2 signaling (**Fig. 1D**).

Protein–protein interaction assays in yeast and in planta show physical interactions between SMAX1 and PHYB, but the exact mechanism of how SMAX1 could regulate PHYB action is not yet clear (Park et al. 2022). The stability of both PHYB and PIF4 is not altered in *smax1*; however, the expression of PIF4-regulated *YUCCA8* (*YUC8*, an auxin biosynthetic gene induced by high-temperature stress; Sun et al. 2012) is significantly reduced. Together with enhanced *YUC8* induction in *kai2* and reduction in *smax1*, this points to SMAX1 somehow counteracting PHYB inhibition of PIF4, probably via another protein (Park et al. 2022). Unraveling the identity of this protein could be interesting for future research.

### Shade avoidance

Dense canopies raise the need for plants growing underneath to exercise adaptive responses to maximize light capture. The filtering of light by canopy leaves results in a decrease in the ratio of red to far-red wavelengths in the light reaching lower leaves and plants in the understorey. This triggers morphological adaptations cumulatively called shade avoidance responses (Tao et al. 2008, Hersch et al. 2014). Shade-intolerant plants exhibit elongation of hypocotyls, petioles and stems and an impediment in leaf development (Franklin 2008, Ciolfi et al. 2013). These morphological changes can come at a cost of yield which makes shade avoidance undesirable for densely sown crops.

KAI2 signaling negatively regulates the shade avoidance response in *Arabidopsis*. In fact, *kai2, max2* and lines overexpressing *SMAX1* show significantly increased hypocotyl growth, leaf area reduction and petiole length under shading, whereas lines overexpressing *KAI2* and *MAX2*, KAR2-treated Col-0 plants and *smax1* mutants are inhibited in responding to simulated shade. *d14*, *max3* and *max4* mutants show no change compared to the wild type, suggesting that KAI2 signaling but not D14 signaling contributes to regulating shade avoidance response (Xu et al. 2022).

In agreement with *KAI2* regulating auxin abundance in hypocotyls during the dark-to-light transition (Hamon-Josse et al. 2022), hypocotyl growth in response to shade has been linked to over-accumulation of auxin as a result of increased abundance of auxin transport proteins PIN3 and PIN7 also in shade avoidance (Xu et al. 2022) (**Fig. 1E**). This suggests that KAI2 signaling may generally regulate auxin distribution and signaling in several responses to environmental conditions.

### Response to drought

Climate change and shortage of fresh water to irrigate farms often result in farms plagued by drought. Thus, unraveling the molecular mechanisms underlying tolerance has become very important to enable breeding drought-resistant crop varieties (Hu and Xiong 2014).

KAI2-mediated signaling plays a role in drought stress resistance (**Fig. 1F**). Initially, this has been shown for *max2* mutants (Bu et al. 2013, Ha et al. 2014), but the link with either KL or SL signaling was not clear. Li et al. (2017) showed that the *Arabidopsis kai2* mutant is hypersensitive to drought. It displayed increased stomatal opening and cell membrane damage resulting in increased water loss upon drought. In addition, *kai2* was found to be less sensitive to ABA, an important hormone regulating drought tolerance (Osakabe et al. 2014, Li et al. 2017). Additionally, *kai2* showed impaired accumulation of anthocyanins and a reduced leaf cuticle which contributes to drought hypersensitivity. Some of these phenotypes were co-regulated by D14 albeit with varying levels of D14 contribution (Li et al. 2017, 2020). Consistent with a major contribution of KAI2, *Arabidopsis smax1smxl2* mutants show increased resistance to drought (Feng et al. 2023). The double mutant exhibits slower leaf water loss, most likely a result of decreased leaf cuticular permeability and stomatal aperture and increased ABA responsiveness. Additionally, *smax1smxl2* accumulates less reactive oxygen species (ROS), possibly from increased *GLUTATHIONE PEROXIDASE3* (*GPX3*) and *GPX7* expression. Furthermore, increased root hair length and root-to-shoot ratio in *smax1smxl2* compared to the wild type demonstrate a stronger drought tolerance response (Carbonnel et al. 2020a, Feng et al. 2023). In *B. distachyon* however, *Bdkai2* experienced only a minor increase in water loss compared to the wild type, but the wild type *BdKAI2* could complement the *Atkai2* water loss phenotype depending on the expression levels of the transgene (Meng et al. 2022). Thus, the need for *KAI2* in drought stress response may differ among species.

### Osmotic stress tolerance

Similar to high-temperature stress, *Arabidopsis kai2* seeds germinate less under osmotic stress compared to the wild type, whereas germination of wild-type seeds subjected to osmotic stress is inhibited by KAR_2_ treatment (Wang et al. 2018). This suggests that KAI2 also functions in osmotic stress tolerance confirming its role in mediating germination only under favorable conditions (**Fig. 1G**). GA treatment can rescue seed germination in *kai2* under osmotic stress (Wang et al. 2018) but whether it can overpower the inhibitory effects of KAR_2_ has not yet been assessed.

KAI2 continues to be important also post-germination for tolerance to osmotic stress. *kai2* plants exhibit increased growth impairment, decreased survival rates and reduced biomass compared to wild-type *Arabidopsis* under salt stress (Mostofa et al. 2023). How KAI2 enhances the chances of survival under these conditions has been investigated by Mostofa et al. (2023). They demonstrate that *KAI2* improves the ability of the plant to regulate shoot Na^+^ homeostasis, possibly by positive regulation of ion transporter genes such as *SALT OVERLY SENSITIVE* and *HIGH-AFFINITY POTASSIUM TRANSPORTER 1;1*. Furthermore, wild-type plants exhibit less oxidative stress–induced damage compared to *kai2*. This can result from higher expression and stronger activity of antioxidant enzymes like superoxide dismutase, catalase and ascorbate peroxidase among others in the wild type compared to *kai2*. In addition, genes involved in SL, ABA, jasmonic acid and salicylic acid biosynthesis and signaling are induced under salt stress in the wild type, whereas the induction is hampered in *kai2* (Mostofa et al. 2023). This *KAI2*-dependent activation of other phytohormone pathways enhances the arsenal available to survive stressful conditions.

In the future, it will be interesting to decipher how the perception of abiotic stress activates KAI2 signaling. Since KAR treatment can enhance tolerance to various stresses (Wang et al. 2018, Shah et al. 2020, 2021), it is possible that stress signaling regulates KL biosynthesis.

### Phosphate starvation responses

Phosphorus is a vital macronutrient for plant growth and development. The slow release of inorganic phosphate (P_i_) from its soil reservoirs makes it poorly available to plants (Shen et al. 2011). One of the ways plants respond to phosphate shortage is by increasing root hair length allowing more efficient foraging of the accessible soil volume (Lynch 2011). KAI2 contributes to the root hair elongation response of *Arabidopsis* to low P_i_ (Villaécija-Aguilar et al. 2022). Mutation of *kai2* and *max2* results in a dampened root hair elongation response to reduced P_i_ concentration in growth media, while *smax1 smxl2* mutants show increased root hair length even at high phosphate. *kai2, max2* and *smax1 smxl2* roots can still sense phosphate starvation, evident from induced expression of P_i_ starvation marker genes *PHOSPHATE TRANSPORTER 1;4* and *INDUCED BY PHOSPHATE STARVATION 1*, and a slight root hair growth response to variations in P_i_ concentrations, which is though much weaker than in the wild type. Additionally, P_i_ starvation induced the expression of *KAI2, MAX2* and *DLK2* in a *PHOSPHATE STARVATION RESPONSE 1 (PHR1)-* and *PHR1-LIKE* (*PHL1)*-dependent manner (both being central transcription factors in phosphate starvation signaling; Paries and Gutjahr 2023) (**Fig. 1C**), suggesting that phosphate signaling regulates the abundance of KL receptor. Thus, *KAI2* is essential for root hair growth responses to phosphate starvation stress. It will be exciting to learn in the future, whether KAI2 signaling also participates in regulating deficiency responses to other important nutrients.

## Regulation of Biotic Interactions

### Letting friends in

A majority of land plants tackle nutrient deficiency by forming mutualistic relationships with Glomeromycotina fungi, termed arbuscular mycorrhiza (AM). The fungi colonize the root cortical cells forming highly branched hyphal structures, the arbuscules, which are sites for the transfer of nutrients to the plant such as phosphorus and nitrogen. In return, the fungus receives carbohydrates and lipids (Keymer and Gutjahr 2018, Wipf et al. 2019). Each step in root colonization is precisely regulated at the cellular and molecular level (Gutjahr and Parniske 2013).

KAI2 plays an important role for colonization of rice roots. Deletion or mutation of *D14L* (rice ortholog of *Arabidopsis KAI2*), results in inability of AM fungi to penetrate the root epidermis (Gutjahr et al. 2015; Choi et al. 2020) (**Fig. 1I**). *Osd14l* is impaired in gene expression responses to germinating spore exudates, suggesting that *OsD14L* functions upstream of or in perception of fungal signaling molecules, or in transcriptional regulation of the response to these. A similar phenotype has been reported for *Petunia kai2a, Brachypodium kai2*, Barley *d14l* and *Medicago kai2a kai2b* (Liu et al. 2019, Meng et al. 2022, Li et al. 2022b). Consistently, rice and *Medicago smax1* exhibit a significant increase in colonization levels (Choi et al. 2020, Li et al. 2022b). However, *KAI2* is not required for AM development in *Marchantia paleacea* (Kodama et al. 2022), suggesting that KAR signaling may have been wired to AM signaling later in evolution in the vascular plant lineage.

Although no direct targets of SMAX1 acting in AM development have yet been determined, Choi et al. (2020) and Li et al. (2022b) place KAI2 signaling upstream of the regulatory network promoting AM development. Choi et al. (2020) reported transcriptional upregulation of SL biosynthesis pathway genes in non-inoculated *smax1* roots. This included GRAS transcription factor *NODULATION SIGNALING PATHWAY 2* (*NSP2*), known to be required for the expression of SL biosynthesis genes (Liu et al. 2011), beta carotene isomerase *DWARF 27* (*D27*), *CAROTENOID CLEAVAGE DIOXYGENASE 7* and *8* (*CCD7* and *CCD8*), which are required for the production of SL precursors, and *CYP711A2*, which is involved in the production of orobanchol (Zhang et al. 2014). An increase in the production of a rice canonical SL 4-deoxyorobanchol could also be observed in the roots. If suggestions of a possible negative impact of SL sensing on AM colonization are true (Yoshida et al. 2012, Gutjahr et al. 2015, Li et al. 2022b), then a negative feedback loop could be assumed, which could be addressed by observing colonization levels in *smax1 d53* double mutants. Nonetheless, *smax1* and *d53* phenotypes are largely mutually exclusive (Soundappan et al. 2015).

The list of genes upregulated in the *smax1* mutant also contains regulators of root colonization which are part of the so-called common symbiosis signaling network, which also operates in root nodule symbioses (RNSs), such as *SYMBIOSIS RECEPTOR-LIKE KINASE* (*SYMRK*), *Ca^2+^/CALMODULIN-DEPENDENT PROTEIN KINASE* (*CCaMK*) and *CYCLOPS* (Oldroyd 2013), and receptor-like kinases, such as *LysM-RECEPTOR-LIKE KINASE 2* (*LysM-RLK2*) [*NOD FACTOR RECEPTOR 5* (*NFR5*)], which may be involved in the perception of fungal lipochitooligosaccharide (LCO) signals (He et al. 2019) (**Fig. 1I**). The perception of fungal LCOs by plants triggers a characteristic calcium spiking signal. This signal initiates a cellular development program in root cells to accommodate AM fungi (Gutjahr and Parniske 2013, Feng et al. 2019). Li et al. (2022b) observed that this spiking was abolished in the rice *d14l* mutant. Furthermore, barley *d14l* was impaired in the induction of *RECEPTOR-LIKE KINASE 10* (*RLK10*) (barley homolog of *NFR5*) and KAR treatment could induce *RLK10* in a *D14L*-dependent manner (Li et al. 2022b). Together with the reduced SL exudation, which is required in normal amounts to activate the fungus, this may explain the impaired root colonization of *kai2* mutants.

Consistent with the role of common symbiosis signaling in RNS and the role of SMAX1 in suppressing the expression of common symbiosis genes, the symbiosis between nitrogen-fixing rhizobia and legumes may also be affected by KAI2 signaling. RNAi of KAI2 and MAX2 in soybean led to a slight reduction in nodule formation; however, this was also the case for D14 knockdown, although to a lesser extent (Ahmad et al. 2020). Knockout mutants will be required to clearly define the role of KAI2- and D14-mediated signaling in RNS development.

### Keeping foes out

Phytohormone signaling and cross-talk thereof play essential roles in mediating plant responses to pathogen attacks and KAI2-mediated signaling is no exception. *Arabidopsis kai2* and *max2* mutants are more susceptible to the hemibiotrophic bacterium *Pseudomonas syringae* pv. *tomato* (*Pst*), whereas surprisingly *smax1* as well as *smxl6/7/8* can suppress the susceptibility phenotype of *max2*. However, the resistance phenotype is specific to *KAI2* signaling, and the authors suggest that SMXL6/7/8 are somehow required for SMAX1 function (Zheng et al. 2023). The susceptibility phenotype of *kai2* and *max2* was attributed to larger stomatal openings and an impaired ability to close stomata in response to pathogen attack leading to unconstrained bacterial entry into the apoplast (Piisilä et al. 2015, Zheng et al. 2023) (**Fig. 1H**).

Beyond the physical barrier, *max2* plants are compromised also at a molecular level. Transcriptional analysis of the mutant showed reduced expression of genes involved in salicylic acid–dependent resistance such as *ISOCHORISMATE SYNTHASE 1* (*ICS1*), *SYSTEMIC ACQUIRED RESISTANCE DEFICIENT 1* (*SARD1*), *NON-RACE-SPECIFIC DISEASE RESISTANCE 1* (*NDR1*), *PHYTOALEXIN DEFICIENT 4* (*PAD4*), *TGA1A‐RELATED GENE 3* (*TGA3*) and *NAC TRANSCRIPTION FACTOR-LIKE 9* (*NTL9*) to list a few in the initial stages of the infection, and this was rescued by *smax1* (Zheng et al. 2023) (**Fig. 1H**). SA-dependent defense response is essential for the establishment of local and systemic resistance primarily against biotrophic and hemibiotrophic pathogens (Spoel et al. 2007). SA treatment can enhance disease resistance in *max2* but not to the level of the wild type, suggesting that SA acts downstream to KAI2 signaling (Zheng et al. 2023). Still, no direct link between the two pathways is yet known and will be interesting to pinpoint in future research.

While KAI2 signaling acts against *P. syringae* in *Arabidopsis*, it does not appear to contribute to resistance against fungal pathogens *Magnaporthe oryzae* (in rice roots) and *Botrytis cinerea* (in *Arabidopsis* leaves) (Gutjahr et al. 2015, Piisilä et al. 2015). Perhaps its role is limited to defense against pathogens that enter the plant through stomata. Further research with a range of pathosystems will reveal the breadth of KAI2 functions in pathogen defense.

## Conclusion and Outlook

Since the discovery of the KAR receptor KAI2 (Waters et al. 2012), our knowledge of KAI2-mediated signaling has rapidly expanded. It has been firmly established that KAI2 signaling is required for normal plant growth and development and plays a central role in responses to abiotic conditions and interactions with microbes, especially with AM fungi. Moreover, first evidence for KAI2 signaling and cross-talk with other phytohormone signaling pathways is emerging. However, equally large gaps in our knowledge remain yet to be filled.

We still lack knowledge on the identity of KL and enzymes involved in its biosynthesis. Targeted in silico modeling led to a sesquiterpene lactone (Rahimi and Bouwmeester 2021), while experimental evidence suggests that the structure of KL might contain a hydrolyzable desmethyl-butenolide ring, without a 4ʹmethyl group (Yao et al. 2021). Desmethyl-GR24*^ent^*^-5DS^ (dGR24*^ent^*^-5DS^) activates KAI2 signaling more strongly in *Arabidopsis*, *B. distachyon* and *M. polymorpha* as compared to GR24*^ent^*^-5DS^ and does not induce signaling via D14 (Yao et al. 2021). Additionally, *Selaginella moellendorffii* KAI2 binds dGR24*^ent^*^-5DS^ in vitro and shows activation in a heterologous system, which otherwise does not occur upon either KAR or GR24*^ent^*^-5DS^ treatment (Waters et al. 2015b, Yao et al. 2021, Meng et al. 2022).

It has been speculated that NSP transcription factors may regulate the KL biosynthesis pathway (Li et al. 2022b). They are required for the expression of genes involved in apocarotenoid biosynthesis. Additionally, the promotion of AM colonization of *M. truncatula* by ectopic expression of *NSP2* requires *KAI2*, and inhibition of colonization in *nsp2 nsp2l* double mutants is suppressed by *smax1* mutation (Li et al. 2022b). It will be interesting to learn in the future if KL is really an apocarotenoid or derived from another biosynthetic pathway.

Despite some indications of KAI2-signaling interactions with other phytohormone pathways, we certainly do not know the whole range of interactions and in most cases, direct molecular connections to these other pathways remain to be uncovered. Particularly, we are missing knowledge about SMAX1 targets. These may differ depending on the scenario regulated by SMAX1. SMAX1 interactomes in different tissues and under different conditions may lead us to these targets. Major exciting questions remain to be answered.

## Data Availability

No new datasets were generated or analyzed in this study.
